# gazeMapper: A tool for automated world-based analysis of gaze data from one or multiple wearable eye trackers

**DOI:** 10.3758/s13428-025-02704-4

**Published:** 2025-06-03

**Authors:** Diederick C. Niehorster, Roy S. Hessels, Marcus Nyström, Jeroen S. Benjamins, Ignace T. C. Hooge

**Affiliations:** 1 Lund University Humanities Lab, Lund University, Lund, Sweden; 2 Department of Psychology, Lund University, Lund, Sweden; 3 Experimental Psychology, Helmholtz Institute, Utrecht University, Utrecht, The Netherlands; 4 Experimental Psychology & Social, Health and Organizational Psychology, Utrecht University, Utrecht, The Netherlands

**Keywords:** Eye tracking, Wearable eye tracking, Mobile eye tracking, Eye movements, Gaze, Data quality, Head-fixed reference frame, World-fixed reference frame, Plane, Surface, Tool

## Abstract

The problem: wearable eye trackers deliver eye-tracking data on a scene video that is acquired by a camera affixed to the participant’s head. Analyzing and interpreting such head-centered data is difficult and laborious manual work. Automated methods to map eye-tracking data to a world-centered reference frame (e.g., screens and tabletops) are available. These methods usually make use of fiducial markers. However, such mapping methods may be difficult to implement, expensive, and eye tracker-specific. The solution: here we present gazeMapper, an open-source tool for automated mapping and processing of eye-tracking data. gazeMapper can: (1) Transform head-centered data to planes in the world, (2) synchronize recordings from multiple participants, (3) determine data quality measures, e.g., accuracy and precision. gazeMapper comes with a GUI application (Windows, macOS, and Linux) and supports 11 different wearable eye trackers from AdHawk, Meta, Pupil, SeeTrue, SMI, Tobii, and Viewpointsystem. It is also possible to sidestep the GUI and use gazeMapper as a Python library directly.

## Introduction

Wearable eye trackers have become more accessible and have improved tremendously in recent years, with features such as plug-and-play recording using devices such as smartphones that fit in your pocket, higher recording frequencies, calibration-free quick setup, and increased robustness. These devices are suited for scientific research that is challenging or infeasible with table-mounted eye trackers, such as studies in sports (Hüttermann et al., 2014, 2018; Hall et al., 2014; Vansteenkiste et al., 2014; Nieuwenhuys et al., 2008; Afonso et al., 2014; Milazzo et al., 2016; Piras et al., 2014; Timmis et al., 2014), tea-making (Land et al., 1999), usability with physical objects (Li et al., 2020; Masood and Thigambaram, 2015; Bergstrom and Schall, 2014), shopping behavior (Gidlöf et al., 2013, 2017), human interaction (Jongerius et al., 2021; Ho et al., 2015; Rogers et al., 2018; Macdonald and Tatler, 2018) and collaboration (Hessels et al., 2023; Lee et al., 2017; Schneider et al., 2016), navigation in urban environments (Kiefer et al., 2014; Koletsis et al., 2017), stair climbing (Ghiani et al., 2023, 2024) and gaze behavior of a teacher in the classroom (McIntyre et al., 2017; McIntyre and Foulsham, 2018), and eye tracking in other complex environments.

**Fig. 1:**
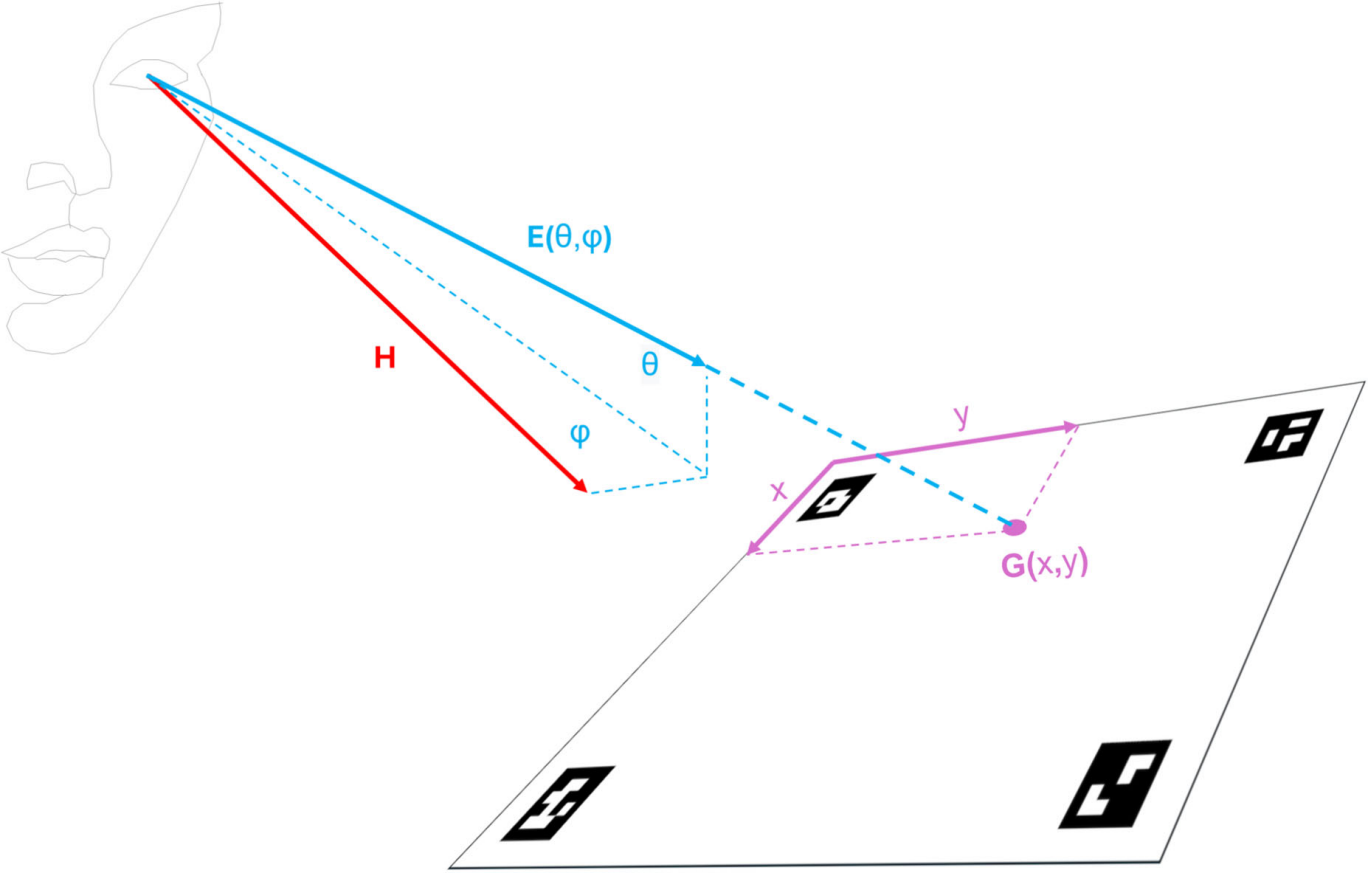
Transformation from eye orientation (in a head-centered reference frame) to a gaze point (in a world-centered reference frame). A wearable eye-tracker delivers the orientation of the eye (*E*, *light blue vector*) with respect to the orientation of the head (*H*, *red vector*). Angles $\theta$ (elevation) and $\phi$ (azimuth) denote the eye-in-head orientation. However, most researchers are interested instead in the gaze position in the world (*G*, the purple intersection point of the extension of the eye orientation vector and the plane, at plane-coordinates *x* and *y*). In gazeMapper, ArUco markers on the plane enable transforming the eye-in-head orientation to a gaze position in a planar reference frame

Despite the breadth of possibilities opened up by the wearable eye tracker form factor, using a wearable eye tracker also has disadvantages. Whereas a remote eye tracker delivers a gaze signal that is referenced to the world (e.g., a computer screen), a wearable eye tracker does not. A wearable eye tracker is fixed to the head of a participant and the eye-movement data it records are therefore expressed with respect to the participant’s head. In this paper, we will call reference frames with respect to a location in the world *world-centered*, while we use *head-centered* to refer to the reference frame for data expressed with respect to the participant’s head (see Fig. 1 below, and Nyström et al. , 2025; Lappi , 2016; Hessels et al. , 2018; Hooge et al. , 2024). While wearable eye trackers can thus very effectively measure *eye movements*, many researchers using these devices are not interested in how the participant’s eyes moves in their head, but instead in gaze locations in the world. Researchers using wearable eye trackers may aim to determine, for instance, what products individuals look at during grocery shopping (Bartels et al., 2018; Otterbring et al., 2016; Gidlöf et al., 2017), where a football player looks while taking a penalty shot (Wood and Wilson , 2011; Noël and Van Der Kamp , 2012 see Belopolsky et al. , 2025; Kurz and Munzert , 2018, for reviews), or where people look while building a Duplo model together (Hessels et al., 2023, 2025a).

Since a wearable eye tracker does not directly report the object or location in the world looked at, determining where a participant looks is an analysis problem. Luckily, a wearable eye tracker recording in most cases also contains a video captured with a scene camera (also called world camera) which provides the required context for interpreting the recorded eye movements. This scene camera, since it is also fixed to the participant’s head, captures a video of a part of the visual world from the participant’s perspective. The video recorded with the scene camera allows tackling the analysis problem of determining *what* a participant looks at in multiple ways.

First, for a human being, what the participant looks at is visible in the scene camera video. The analysis approach for many studies has therefore involved manually mapping gaze to locations or objects in the world using scene camera videos on which the gaze position was overlaid (e.g., Gidlöf et al., 2013; Gidlöf et al., 2017; Land et al., 1999; McIntyre and Foulsham, 2018; Ho et al., 2015; Rogers et al., 2018; Yu and Smith, 2017; Benjamins et al., 2018; Maran et al., 2022; see Hessels et al., 2020; Fu et al., 2024; Franchak and Yu, 2022, for insightful discussions). Manual coding would entail going through the scene video fixation-by-fixation, or even frame-by-frame, and determining for each moment what is looked at, such as “hands of the goalie”. Such manual coding is very time-consuming. For instance, Rogers et al. (2018) noted that coding their gaze data took 62 h (or about 9 min per minute of gaze data), while the manual analysis of Maran et al. (2022) took 830 h for 118 recordings of 5–7 min (about 7 h per recording). Benjamins et al. (2018) further reported that eight human coders on average each took 879 s to code 330 s of data. Manually coding gaze data may thus present a significant bottleneck in performing a study using wearable eye-tracking data. Fortunately, there are also automated solutions for determining what participants look at from wearable eye tracker recordings (see Niehorster et al. , 2025, for an overview) which we will discuss next.

Second, the mapping process for determining what participants look at can be automated by analyzing the content of the scene camera image and determining where, for instance, people or certain target objects are located. Once the positions of objects of interest in the scene camera are known, an automated analysis can then match the gaze data to these object locations to determine whether participants looked at them and for how long. Examples of such approaches are the methods presented by, e.g., Alinaghi et al. (2024); Mercier et al. (2024) (see Niehorster et al. , 2025, for many more).

Third, the gaze position in the external world can be determined by performing a coordinate transformation of the head-centered eye movement data delivered by a wearable eye tracker to a world-based reference frame (Nyström et al., 2025; Lappi, 2016; Hessels et al., 2018; Hooge et al. 2024, see Fig. 1). One way to perform this transformation is by using detectable and recognizable markers within the scene camera’s visual field. Such markers can, for instance, be used to define one or more flat planes in the environment and enable automated transformation of head-centered eye movement data to gaze positions on each of these planes. Examples of such planes include a large television, a table, a mobile phone, a store shelf, a poster, screens and control panels in a nuclear power plant or an aircraft cockpit, a map of France, and a vehicle dashboard. These planes can cover areas much larger than a single computer screen (see, e.g., Faraji et al., 2023; Hessels et al., 2023). Once data is mapped to a plane, one of the hard problems of wearable eye tracking is solved; for instance, standard methods for fixation classification (Hooge et al., 2022b; Andersson et al., 2017; Hein and Zangemeister, 2017) and automated standard Area of Interest (AOI) analysis (Hessels et al., 2016; Holmqvist et al., 2011; Goldberg and Helfman, 2010) using AOIs defined on the plane can now be applied to the data.

Different markers can facilitate the transformation from a head-centered to a world-centered reference frame. For instance, the now obsolete Tobii Glasses 1, introduced in 2010, were equipped with unique infrared markers that could be placed in the environment to define planes. This functionality was used in studies like Dik et al. (2016), which examined the gaze behavior of endoscopists during a colonoscopy session, using markers on the edge of the endoscopy monitor (see also, Weibel et al., 2012). Another (cheaper and more generic) method involves placing visual markers, such as printed markers, in the environment (e.g., Land and Lee, 1994; Pfeiffer and Renner, 2014; Tabuchi and Hirotomi, 2022; Duchowski et al., 2020; Niehorster et al., 2023; Niehorster et al., 2020a; Faraji et al., 2023; Bykowski and Kupiński, 2018; Yang and Chan, 2019; De La Hogue et al., 2024; Munn and Pelz, 2009; Kiefer et al., 2014). There are various types of visual markers (e.g., ArUco markers, Garrido-Jurado et al., 2016; April tags, Wang and Olson, 2016; and ARTag, Fiala, 2005; for overviews, see Kalaitzakis et al., 2021; Jurado-Rodriguez et al., 2023); we use ArUco markers for the tool discussed in this paper since these are both commonly used and easily processed using OpenCV.

No open, eye-tracker agnostic and easy-to-use tools with a graphical user interface (GUI) are available that allow users without advanced computer skills to perform world-based analysis of gaze data from a wearable eye tracker. Although there are many methods for world-based analysis of gaze data (the marker-based techniques are just one type of method amongst many, see Niehorster et al. , 2025, for an overview), almost all published methods (e.g., Alinaghi et al. , 2024; Mercier et al. , 2024; Paletta et al. , 2013; Mardanbegi and Hansen , 2011) have a significant barrier to usage. They require at a minimum that the user is comfortable setting up their own scripting environment (e.g., installing Anaconda to access Python) and can adapt and run the provided code for their setup. It might even be required to implement the published method from scratch using the description provided by the authors. A welcome exception is software from manufacturers like Tobii and Pupil Labs, which can utilize markers or other methods to transform eye-tracking data from a head-centered to a world-centered reference frame. Why then do we here present a tool that can perform head-to-world transformations for gaze data and have written an article about it? After all, the problem has already been solved and implemented in manufacturers’ software. The answer is that, even though the manufacturers’ software is capable of fantastic things, it also has limitations. The manufacturers’ software is often closed-source, making it impossible to check, fix or enhance the implementation. Some manufacturers’ software is hosted in the cloud, and thus requires an internet connection to use it. Manufacturers’ software furthermore, naturally, only supports the manufacturer’s own eye trackers and may thus be of no use if a different eye tracker is used. Further issues may crop up if a manufacturer decides to discontinue a required feature or support for an old eye tracker, or decides to start charging money for software that was previously freely accessible. Here we list some issues and use cases that current corporate software cannot fully address:

1. Suppose a researcher conducts studies in two laboratories with different wearable eye trackers (e.g., Hessels et al. , 2025a). Ideally, a researcher would only have to learn how to use one eye tracker-agnostic analysis tool to work with the data from both eye trackers. This would also enable a researcher who aims to conduct a study with both eye trackers to seamlessly process the data from both devices. Processing the eye-tracking data from both labs using the same analysis pipeline furthermore avoids differences in results that may be caused by the processing software.
2. Replication studies may face challenges if the original software for the eye tracker is no longer available, which is especially likely with older studies (e.g., Dik et al. , 2016; Vansteenkiste et al. , 2014). Since wearable eye trackers are undergoing rapid development, open and eye-tracker-agnostic software can be beneficial in such situations.
3. A researcher conducts a study where two eye trackers (for instance, an old SMI ETG and a new SeeTrue STONE) are used in the study at the same time, for instance, to study collaboration between two people. For such studies, the researcher not only needs to be able to map gaze, it is also required that their analysis software can synchronize the data from the different devices so that they can assess whether and how the behavior of their two participants is temporally coordinated (cf., Hessels et al. , 2023; Hessels et al. , 2025a).
4. The study requires that eye movement data from one eye tracker is mapped into the view of another eye tracker or into that of an overview camera with which a simultaneous recording is made, either for visualization or for analysis purposes.
5. We observed (Hooge et al., 2022a; Hessels et al., 2025a) that the scene camera and eye cameras of wearable eye trackers are not always perfectly synchronized. Therefore, the software would ideally include functionality to solve such synchronization issues.

**Fig. 2:**
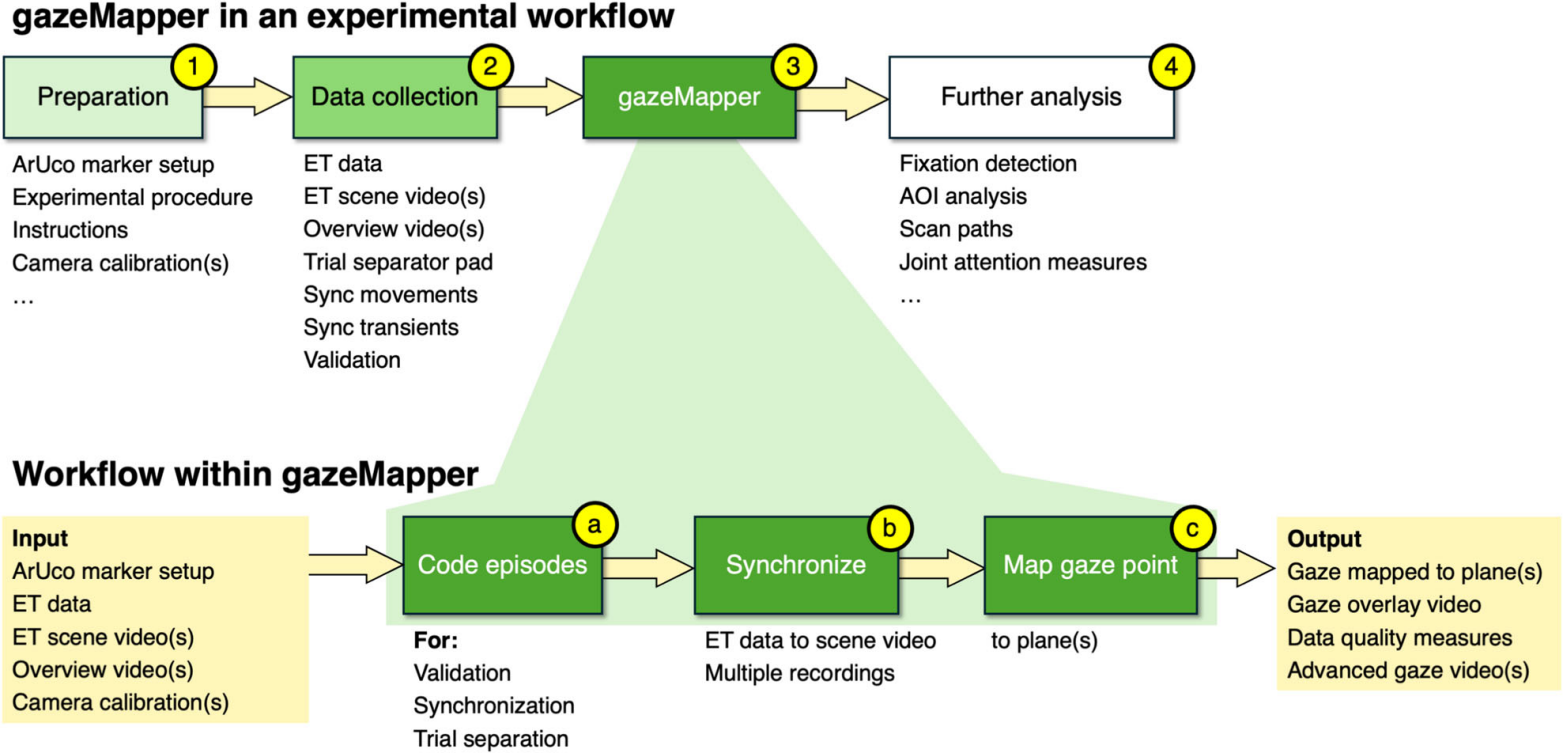
Overview of where gazeMapper fits in an experimental workflow, and of the workflow within gazeMapper. In this figure and caption, ET stands for eye tracking, and AOI for area of interest. *(1) Experiment preparation*. *Mandatory*: ArUco marker setup to delineate plane(s) (see the “Workflow” Section, point 1a and the “Defining planes and mapping gaze” Section). *Optional*: create experimental procedure and instructions for validation using glassesValidator functionality (“Workflow” Section 1a and 2a), for synchronization of multiple recordings, and of the eye tracking data to the scene camera (see Sections “Synchronization of eye movement data and scene camera” and “Multiple recording synchronization” and for trial segmentation (“Workflow” Section, 1c, 2b and 2c). *(2) Data collection*. *Mandatory*: Collect one or more ET data and scene video recordings, and zero or more overview camera recordings. *Optional*: Use the trial separator pad (Fig. 6, “Workflow” Section 1c and 2c) or other prepared method to enable trial separation in step 3a. Execute the VOR synchronization movement procedure to enable removing temporal offsets (“Workflow” Section 2b), the validation procedure to enable determining data quality for the recording (“Workflow” Section 2a), and show synchronization transients to enable synchronizing multiple recordings (“Workflow” Section 1c and 2c). *(3) Workflow within gazeMapper*. The data recorded during step 2, along with the ArUco setup created in step 1 are used as input (“Workflow Section” 3). *(3a) Code episodes*. First, the recorded videos are coded by the researcher using the gazeMapper GUI to indicate which intervals in the recording contain the trials, the validation task, and the synchronization task and transients (“Workflow” Section 5). *(3b) Synchronization*. The coding is used to synchronize the ET data to the respective scene videos (see the “Synchronization of eye movement data and scene camera” Section) and synchronize together multiple recordings (see the “Multiple recording synchronization” Section) and to separate out the individual trials. *(3c) Mapping the gaze point*. The resulting synchronized ET data streams are mapped to one or multiple planes in the world (see the “Defining planes and mapping gaze” Section). The mapped gaze point data, along with several gaze visualization videos and data quality measures (see the “gazeMapper output and further analysis” Section), can be collated and exported from gazeMapper. *(4) Further analysis*. The output from gazeMapper can then be used for further analyses outside gazeMapper, such as fixation detection, AOI analysis, scan path analyses and joint attention measures

This article introduces gazeMapper, a software tool that can map head-centered eye movement data from 11 different wearable eye trackers onto one or multiple planes delineated by ArUco markers or April tags. It furthermore enables synchronizing simultaneously collected gaze data from multiple participants as well as external overview cameras, and has built-in support for determining data quality measures (accuracy and precision) using glassesValidator (Niehorster et al., 2023). Finally, for any recording made with a supported eye tracker, regardless of whether it contains fiducial markers, gazeMapper is able to render a scene video with overlaid gaze marker. gazeMapper does not provide further analysis of the mapped data, such as fixation classification or AOI analysis. See Fig. 2 for an overview of where gazeMapper fits in a wearable eye tracker study workflow, and for an overview of the workflow for using gazeMapper to analyze wearable eye tracker data. gazeMapper comes with a user-friendly GUI that requires only minimal computer skills to use, ensuring that our methods are accessible to researchers from a wide variety of research areas and backgrounds. In this paper, we describe the implementation, workflow, and interface of this software. Note that this paper describes gazeMapper’s functionality and methods at a high level, it is not a manual, nor a step-by-step guide for using gazeMapper. The user who directly wants to get started and try out the tool is referred to the installation instructions and step-by-step walkthroughs provided in gazeMapper’s manual at https://github.com/dcnieho/gazeMapper. Example data on which these workflows can be tried is also available from this repository.

## Tool description

In this section, we will provide an overview of the functionality provided by gazeMapper. We then describe the core methods by which gazeMapper’s functionality is achieved in further detail.

For gazeMapper to be able to process a wearable eye tracker recording, the recording must consist of at least a video recorded from the eye tracker’s scene camera, and a data file with user-calibrated (if required by the eye tracker) gaze positions expressed in the coordinate system of the scene video (i.e., in pixels on the scene video). All the currently supported eye trackers by default deliver the expected gaze positions and scene video. Currently, gazeMapper supports recordings made by the following eleven 11 eye trackers:

- AdHawk MindLink
- Meta Project Aria Generation 1
- Pupil Core
- Pupil Invisible
- Pupil Neon
- SeeTrue STONE
- SMI ETG 1
- SMI ETG 2
- Tobii Pro Glasses 2
- Tobii Pro Glasses 3
- Viewpointsystem VPS 19

If an eye tracker of interest is not supported by gazeMapper, the researcher has three options. First, the researcher is advised to check whether support may have been added in the latest version of gazeMapper. Second, the researcher can contact the first author who may be interested in adding support for the eye tracker to gazeMapper. Finally, gazeMapper can import gaze data and scene videos from any eye tracker if the data has been transformed to gazeMapper’s internal format (a tab-separated text file containing at least a timestamp, and a horizontal and a vertical gaze position for each measurement sample, as documented in the readme at https://github.com/dcnieho/glassesTools). The user can thus enable support for any eye tracker (including internal prototypes under active development) by writing a converter that transforms the eye tracker’s data format to gazeMapper’s internal data format.

A camera calibration (a mathematical description of the distortions due to the camera’s optical system–importantly its lens) for the eye tracker’s scene camera is used when available, but is not required for the mapping of eye movements to plane(s) in the world. At the time of writing, all of the supported eye trackers except the Viewpointsystem VPS 19 either provide the camera calibration of the scene camera as part of the recording, or gazeMapper includes a generic camera calibration provided by the manufacturer that adequately captures the characteristics of the scene camera used in the given eye tracker model. gazeMapper’s functionality for mapping gaze data to the view of external overview cameras also does not require the overview camera to be calibrated if there is no appreciable distortion in the recorded video (i.e., physically straight lines look approximately straight in the video). As such, the end user in most cases does not have to perform a camera calibration to use gazeMapper’s functionality.

**Fig. 3:**
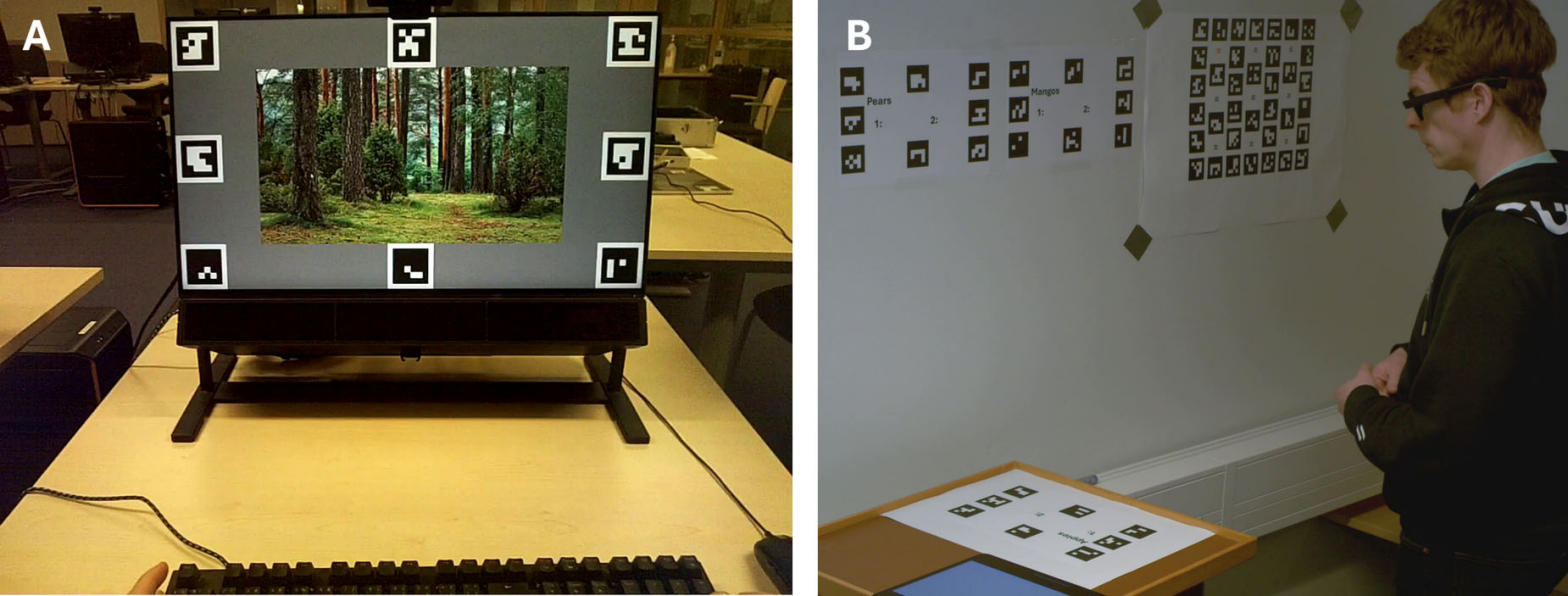
Two examples of planes defined by ArUco markers. **A** The ArUco markers along the edges of the computer screen allow mapping gaze to a position on the screen. **B** A glassesValidator (Niehorster et al., 2023) poster along with three other planes marked “apples”, “pears” and “mangoes”. Both are taken from the example recordings available at https://github.com/dcnieho/gazeMapper

However, if one wishes to use gazeMapper’s built-in support for determining data quality measures (accuracy and precision) using glassesValidator, then the recommendation detailed in the article introducing glassesValidator (Niehorster et al., 2023) to use a calibrated scene camera applies. This is because the calculation of angular (in)accuracy requires information not only about gaze position on the plane, but also the position and orientation from which the observer views the plane. As discussed in Niehorster et al. (2023), determining the viewing position and orientation of the observer requires a calibrated camera.

gazeMapper is available from https://github.com/dcnieho/gazeMapper as a pip-installable Python package that supports Windows, macOS, and Linux. The gazeMapper manual, including detailed installation instructions, is found on the same website. The gazeMapper package can be installed directly from Python using the command python -m pip install gazeMapper.

### Defining planes and mapping gaze

To enable mapping of gaze to a plane in the world, the plane of interest must be localized in the eye tracker’s scene video and it must be possible to extract information about the distance and orientation of the plane from the scene video. In this work, we use arrays of specially designed 2D barcodes called ArUco markers (Garrido-Jurado et al., 2016) for this purpose.^1^ These fiducial markers are easily detected and identified by a computer. See Fig. 3 for two examples of planes defined by ArUco markers. The fiducial markers used to define a plane of interest must have a known size, and the marker array a known spatial configuration (known marker positions and orientations). Then, if some of the fiducial markers defining a plane are fully in view (by default at least three markers), the transformation function for mapping eye movements to the plane can be determined. Using fiducial markers such as ArUco markers is a common approach for determining viewing positions with respect to a plane (see, e.g., Santini et al. , 2017; MacInnes et al. , 2018; Niehorster et al. , 2020a; Niehorster et al. , 2023; Duchowski et al. , 2020, and the Pupil Labs surface tracker).

gazeMapper implements two different methods for transforming eye movements to gaze on a plane using the fiducial markers. First, gazeMapper can directly estimate the homography transformation that maps coordinates in the scene video to coordinates on the plane. If there is no appreciable lens distortion in the eye tracker’s scene video, this transformation can be performed without a calibration of the scene camera. The interested reader is referred to MacInnes et al. (2018) and Niehorster et al. (2020a) for more information about this method. Second, if the eye tracker’s scene camera is calibrated, the fiducial markers allow determination of the scene camera’s pose (location and orientation) with respect to the plane using Perspective-n-Point problem solvers (Wang et al., 2018; Terzakis and Lourakis, 2020; Zheng et al., 2013; Lu et al., 2000). Given the scene camera pose, gaze position on the plane can be determined by intersecting the ray specified by the gaze position on the scene camera image with the plane. Further information about this method is provided in Niehorster et al. (2023).

gazeMapper can also perform the inverse transformation, that is, mapping gaze positions on a plane to pixel locations in a camera. This is used, for instance, when drawing gaze data of one participant on the scene video from another participant, or when drawing gaze data on the view of an overview camera. The same two methods as for the video-to-plane transformation can be used, and the same caveats regarding camera calibration as discussed in the previous paragraph apply.

### Synchronization of eye movement data and scene camera

In the types of unconstrained settings typically targeted with wearable eye trackers, many gaze movements are executed using a tightly temporally coordinated combination of head movements and eye movements (see, e.g., Hooge et al. , 2024; Fang et al. , 2015). Even during fixation, eye movement continually compensates for head movement to maintain a stable gaze position in the world (Kothari et al., 2020; Collewijn et al., 1981; Land and Tatler, 2009; Angelaki, 2004). Therefore, for accurate determination of gaze position in the world, it is critical that the eye movement data delivered by the eye tracker is accurately synchronized to the video recorded by the eye tracker’s scene camera, which undergoes continuous motion as the head moves. We have previously observed that this is not always the case (Hooge et al., 2022a); for example, in Hessels et al. (2025a) we found that the eye movement data had a median time lag of 0.07 s (range: [0.02, 0.11] s) with respect to the scene camera feed for one of the eye-tracking systems.

gazeMapper includes functionality for synchronizing eye movement data with the scene video. Our method, following a suggestion by Matthis et al. (2018, see also Hooge et al. , 2022a; Hooge et al. , 2024), relies on the vestibulo-ocular reflex (VOR), which keeps the eyes fixated on a target in the world while the head is moving. This is possible because VOR has a very low latency (about 10 ms during sudden externally-imposed head rotations, Aw et al. , 1996; Collewijn and Smeets , 2000; and essentially zero during continuous or self-generated head rotations, e.g., King , 2013; Collewijn et al. , 1983; Leigh and Zee , 2015).

**Fig. 4:**
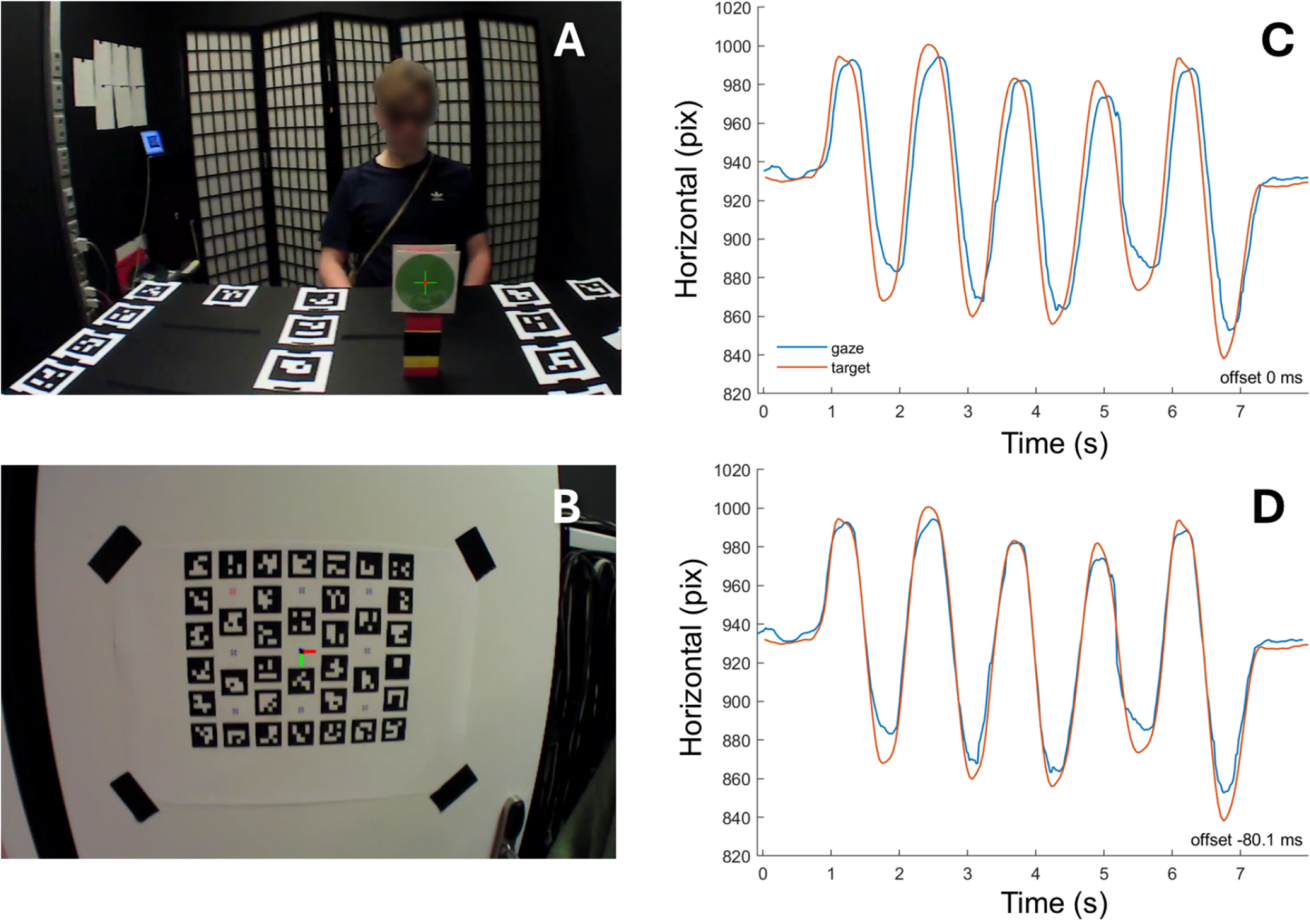
Synchronization of eye movement data to the eye tracker scene camera. **A** Participants are asked to fixate the center of a green dot while oscillating their head. The center of the green dot is detected by a custom function that was configured to run using gazeMapper, and the detection result is indicated by the green cross with a red dot at the center. This is the setup used by Hessels et al. (2023). **B** The center of a gazeMapper plane defined with ArUco markers is used as the fixation target and detected using built-in functionality of gazeMapper. The detected center is indicated by the meeting point of the *red* and green axes. This is the setup used by Hessels et al. (2025a) and in the example recordings available at https://github.com/dcnieho/gazeMapper. **C** Data from a Tobii Pro Glasses 3 during horizontal head oscillations (taken from Hessels et al. , 2025a). As can be seen, the movement of the gaze is very similar to the movement of the target in the scene camera, but temporally offset. This indicates that there is a synchronization problem. **D** The same data as in panel **C**, but manually shifted to synchronize the eye movement data with the scene camera. In this case, an offset of 80.1 ms was applied to achieve accurate synchronization

**Fig. 5:**
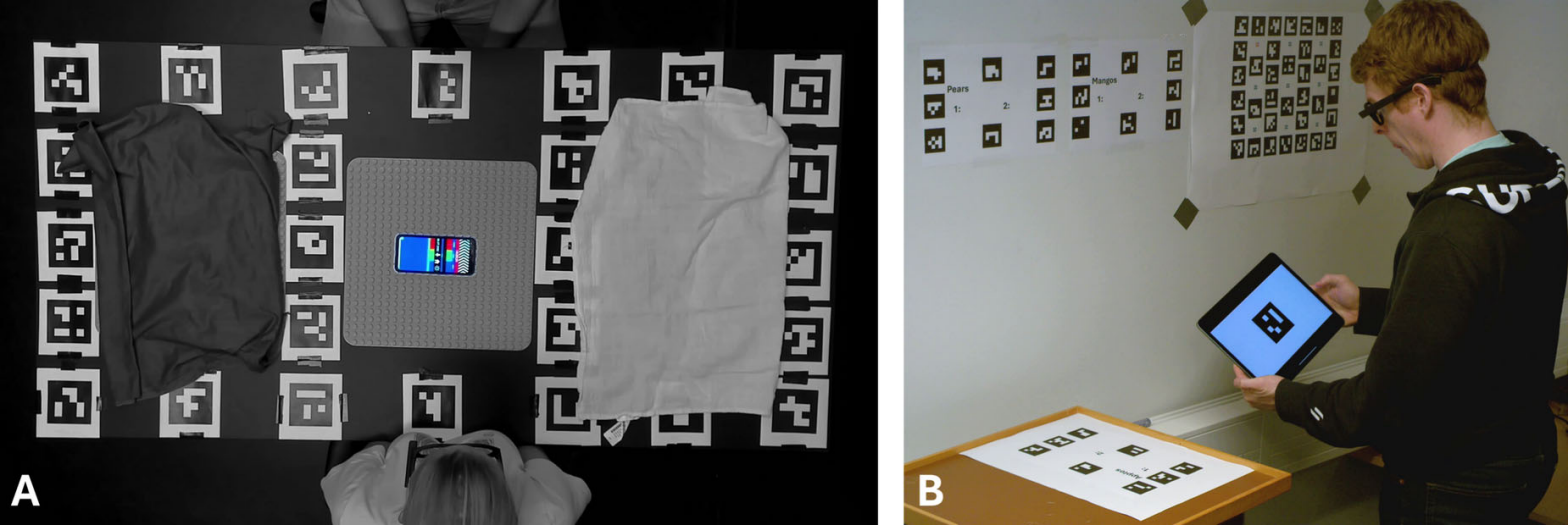
Synchronizing multiple recordings. **A** a digital clapperboard as used by Hessels et al. (2023) is shown on a mobile phone placed in the middle of the table. **B** ArUco marker flashed on a tablet as used in example recording 3 available at https://github.com/dcnieho/gazeMapper (frame taken from the overview camera used in the example recording)

gazeMapper’s method for eye movement and scene camera synchronization makes use of this well-established ability to maintain fixation on a world-fixed target while moving the head using the following procedure. The participant is asked to maintain fixation of a target (see Figs. 4A and 4B for two example fixation targets) while slowly (at about 0.5 Hz) making five horizontal head oscillations (as in shaking their head to communicate “no”) followed by five vertical head oscillations (nodding “yes”). When the participant performs this task, we can assume that they will make eye-in-head movements that are in perfect counter-phase to the head oscillations to maintain gaze on the target. Therefore, if there is no latency between the eye tracker data and the scene camera, we should observe the gaze position to remain fixed on the target in the scene video. If, however, there is latency, the fixation target and the gaze point provided by the eye tracker will move similarly, but appear out of phase. The roughly sinusoidal movements of both the target in the scene video and of the gaze position on the scene video due to the participant’s rhythmic head rotations allow to easily check for synchronization. If the two signals are not in sync, peaks in the roughly sinusoidal signals do not line up (Fig. 4C). Synchronization can be manually corrected using an interface in gazeMapper (see Fig. 8B) by shifting the gaze signal forward or backward in time using the mouse or the arrow keys on the keyboard until its peaks coincide with the peaks in the target signal (Fig. 4D).

While the functionality for synchronizing eye tracker data to the scene video is an optional component of the gazeMapper workflow, we strongly recommend always including the VOR task in an eye-tracking recording. This allows verifying the correct operation of the eye tracker, and to correct synchronization issues if there are any.

### Multiple recording synchronization

When performing joint recordings of multiple participants, or recordings that besides an eye tracker also include overview cameras, it is necessary to synchronize these recordings into a common time base. When recording with multiple devices, two problems may occur. Firstly, there is likely an offset between the starting points of the recordings, which would be visible as a shift in time between the recordings when viewing them side-by-side. Secondly, since the electronic clocks in the computer, camera or eye tracker are prone to drift with respect to each other (Zhao et al., 2008; Jiang et al., 2019; Goudeseune and Kowitz, 2004), recordings that are synchronized at one point in time may become desynchronized over time. gazeMapper’s synchronization functionality allows correcting for these two phenomena. There are no theoretical limitations on the number of eye tracker and overview camera recordings that can be synchronized and analyzed together. The largest recording sessions for which we have used gazeMapper consisted of two eye trackers and one overview camera (Hessels et al. , 2023; Hessels et al. , 2024; Hessels et al. , 2025a, and see also example 3 available at https://github.com/dcnieho/gazeMapper).

Synchronization of recordings is done by means of coding the occurrence of visual transients in the scene video or the overview camera video. These transients can, for instance, be a virtual clapperboard (Fig. 5A), or an ArUco marker that is flashed on a screen (Fig. 5B). To allow for accurate synchronization, it is critical that the visual transient is abrupt and that it is visible to all cameras at the same time. The timepoints at which these transients occur are either manually coded by the researcher for each video or, if flashed ArUco markers are used as synchronization transients, can be automatically detected and coded in the videos by gazeMapper. When the timepoint at which a visual transient occurred is coded for all the recorded videos, the video timestamps (and eye movement data timestamps, if available) for these recordings can be aligned to a single recording, called the reference recording. When more than one visual synchronization transient is coded for the recordings, this furthermore allows estimating differences between the videos in the rate at which time passed. This additionally allows correcting for clock drift by scaling the timestamps so that the rate at which time passes for all recordings equals that of the reference recording. If such correction for clock drift is wanted, it is recommended to space the synchronization transients sufficiently in the recording, for instance, placing them at the beginning and end of the recording (see example 3 available at https://github.com/dcnieho/gazeMapper).

## Workflow

The workflow for performing a recording and analyzing it using gazeMapper depends on the specifics of the study. Here we discuss what to consider during each step of the workflow, from recording preparation to analysis. Notes are included for researchers who wish to include additional eye trackers or overview cameras. gazeMapper includes a graphical user interface (GUI, see Fig. 7) to enable processing recordings without needing to know how to program. It is, however, also possible to call all of gazeMapper’s functionality directly from Python scripts without needing to use the GUI. The interested reader is referred to gazeMapper’s manual for a complete overview of gazeMapper’s programming interface. Data for three different example experiments, along with example output from gazeMapper for these three examples, are available from https://github.com/dcnieho/gazeMapper/tree/master/example_data.

**Fig. 6:**
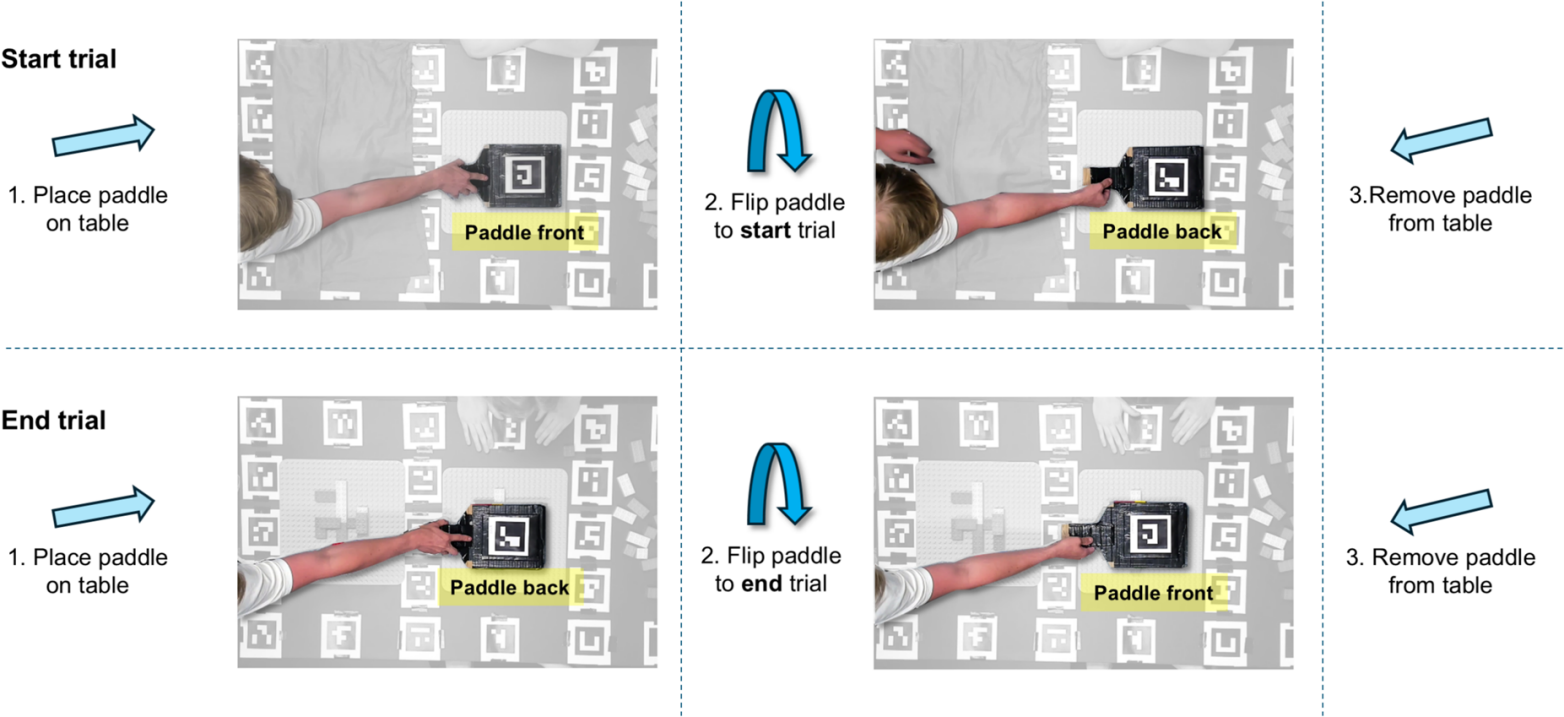
Trial segmentation. *Top row*: The beginning of a trial is marked by placing a paddle containing ArUco markers on the table and then flipping it from one side to the other, exposing the markers in rapid succession to the camera. *Bottom row*: The end of a trial is marked by placing the paddle on the table and flipping it in the reverse order. This method was used by Hessels et al. (2023, 2025a)

**Fig. 7:**
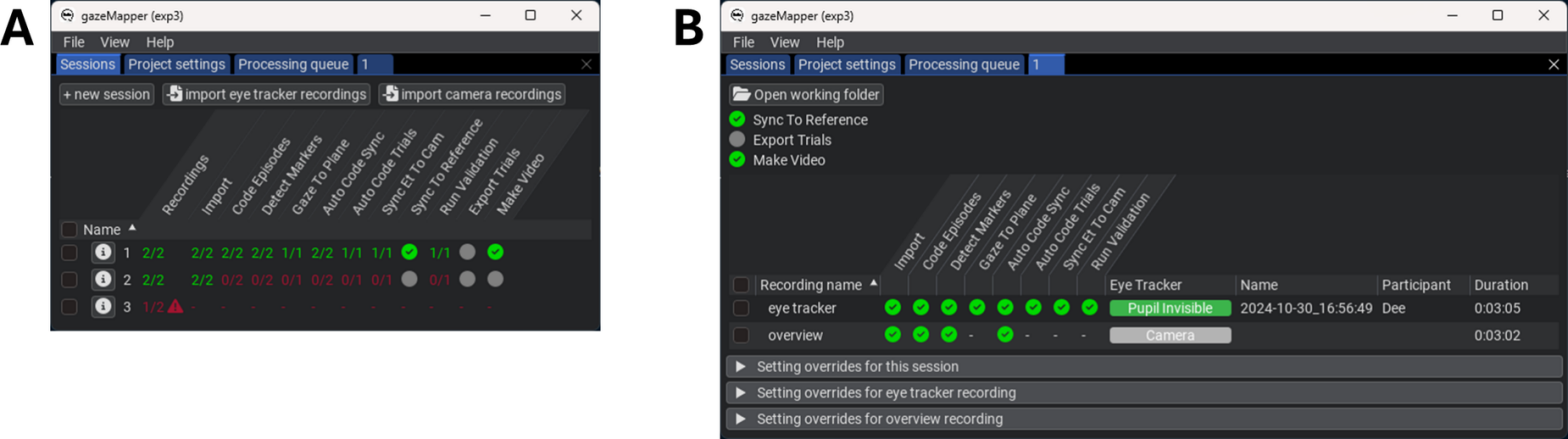
Screenshots of the gazeMapper GUI. **A** The main screen with three sessions. The first session is fully processed as indicated by the green status for the actions. For the second session, both required recordings are imported but no further processing has been done. For the third session, only one of the required two recordings has been imported. **B** Interface to view details of a single session

**Fig. 8:**
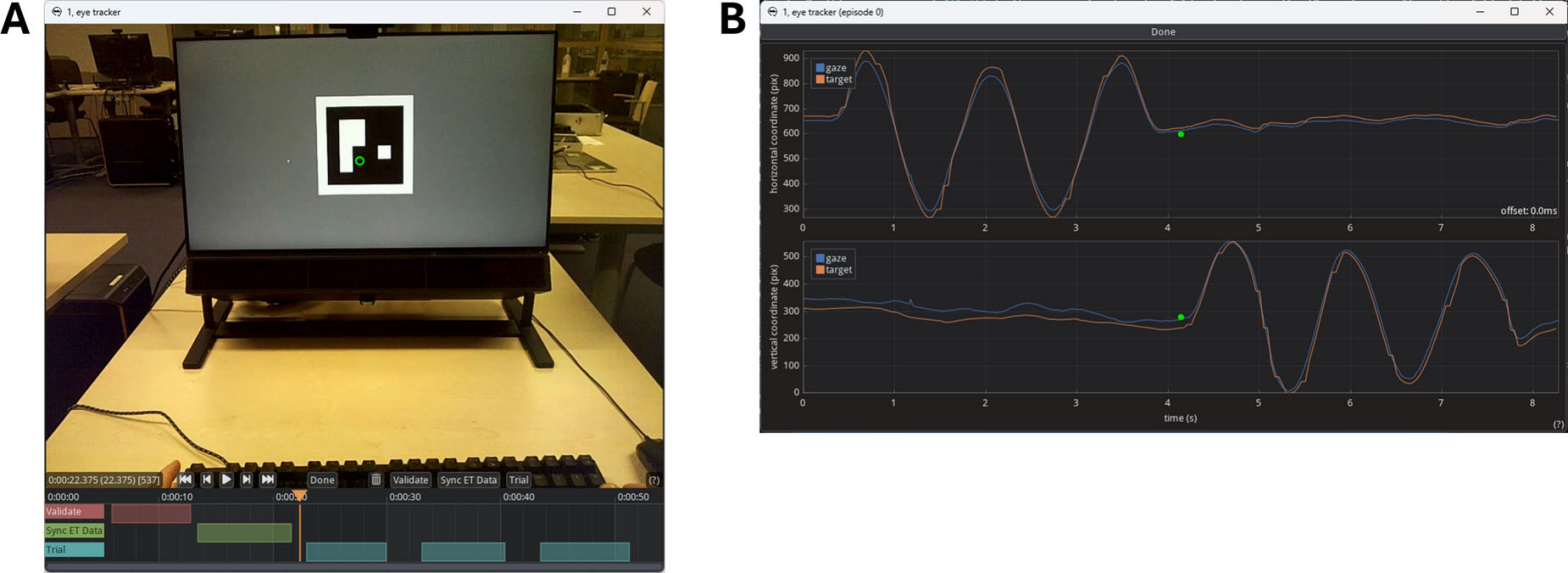
gazeMapper’s coding GUIs. **A** The video coder GUI. Gaze is shown on the scene video (*green circle*) and the coded episodes are indicated in the timeline underneath the scene video. **B** Interface for checking and adjusting synchronization of the eye movement data to the scene video. Shown are the positions of the fixation target and of gaze on the scene camera

The workflow in gazeMapper consists of the following steps:

1. *Preparation*. Before recording, the researcher prepares the recording space by defining one or multiple planes for gazeMapper to map eye movements to. They may furthermore, as required, prepare for the automated determination of data quality; synchronization of the eye movement data to its scene video; trial starts and ends; and camera synchronization transients. They do so as follows:
  - *Define planes*. Planes to map eye movements to are defined by placing an array of ArUco markers of known size on a flat surface in a known spatial configuration (known marker positions and orientations). Although technically only a few markers are needed to enable mapping of eye movements to a plane, we recommend defining a plane using a large number of markers for robustness against potential issues such as bad quality of the video recording or occlusion of markers due to participant behavior. For robust and accurate performance of gazeMapper it is essential that every marker is unique (i.e., used only once across all planes) and that its size, position and orientation are precisely known (ideally at mm resolution).
  - *Data quality assessment*. Data quality should be reported in studies using an eye tracker (Dunn et al., 2023). For wearable eye tracker recordings, data quality (i.e., accuracy and precision, c.f., Niehorster et al. , 2020b; Holmqvist et al. , 2011) can be estimated using a glassesValidator poster (Niehorster et al., 2023). We therefore recommend that the researcher prepares such a poster and includes it in their recordings. When using the glassesValidator poster, the researcher should ensure that none of its markers are used in any of the other planes. The glassesValidator poster can also be used by gazeMapper to assess whether the eye movement data delivered by the eye tracker is synchronized to its scene video camera (cf., Fig. 4B) and to resolve inaccurate synchronization if it is observed.
  - *Trial segmentation and multiple recording synchronization*. gazeMapper is furthermore able to automatically segment the recording into trials and to automatically detect synchronization transients by detecting when additional single ArUco markers are visible to a camera. Should the researcher want to make use of this functionality, they should prepare a method for presenting these markers, such as a paddle with ArUco markers glued on either side for denoting trial starts and ends in the recorded video (c.f., Hessels et al. , 2023; see Fig. 6) or an animated PowerPoint presentation or video running on a tablet to present the synchronization marker (Fig. 5B).
2. *Conducting a recording*. During recording, no special instructions to the participant are needed for mapping eye movements to a plane. However, the use of the glassesValidator poster and ArUco markers for trial segmentation or multiple-recording synchronization does require specific handling by the operator.
  1. *Data quality assessment*. When using the glassesValidator poster, the instructions from Niehorster et al. (2023) apply. They wrote: “The operator positions the participant in front of the glassesValidator poster. An easy method for positioning the participant is to ask them to stretch their arm out straight forward and stand at a distance where their fist touches the poster. The operator then issues the following suggested instructions: ‘look at the nine fixation targets in reading order for 1 s each. Start with the top-left (red) target. When looking at the fixation targets, keep your head as still as possible, move only your eyes.’ These verbal instructions could be accompanied by pointing at the fixation targets in the desired looking order to further help the participant to follow the instructions.”
  2. *Assessing synchronization between eye-tracking data and scene camera*. If using the glassesValidator poster to assess whether the eye movement data delivered by the eye tracker is synchronized to its scene video camera, the participant is positioned in front of the glassesValidator poster using the same fist method as above. They are then issued the following instructions: “look at the center fixation target on the poster and keep looking at it while continuously moving your head like shaking ‘no’ five times. After that, while you keep looking at the fixation target, continuously move your head like nodding ‘yes’ five times.” We recommend that the operator models the expected behavior after these verbal instructions so that the participant has a reference for the rate at which and the manner in which to move their head. We recommend that, during a pilot, operators try out various speeds and movement amplitudes themselves to get acquainted with what works for their setup. As a starting point, they may opt for movement at about 0.5 Hz with an amplitude of about 40° (i.e., $20^{\circ }$ in each direction).
  3. *Trial segmentation and synchronization of multiple recordings*. If using ArUco markers to delineate trial beginnings and ends, the operator should instruct participants to face the ArUco markers and look at them to ensure that they are visible in all camera feeds. Similarly, for synchronization of multiple recordings, the operator must ensure that the visual transient used is visible in all camera feeds. For synchronization, we furthermore recommend displaying a visual transient both near the beginning and near the end of the recording session so that any clock drift, if present, can be compensated for.
3. *gazeMapper configuration*. Before a recording session can be analyzed in gazeMapper, a gazeMapper project must be created and configured. gazeMapper is extensively configurable; the reader is referred to the manual for a description of all settings. The following items likely have to be configured:
  - *Recording sessions*. In gazeMapper, recordings are organized into recording sessions. The researcher must define what recordings make up a session. The minimum configuration is a single eye-tracker recording per session. However, a recording session can also consist of simultaneous recordings from multiple eye trackers or additional recordings with overview cameras (Fig. 7). There is no theoretical limit to the number of eye trackers and overview cameras that can be configured to be part of a session.
  - *Planes*. The planes to which eye movements should be mapped.
  - *Individual markers*. If ArUco markers are used to delineate trial beginnings and ends or for synchronization of multiple recordings, the researcher should configure which markers are used for these purposes.
4. *Importing recordings*. To start processing a recording session in gazeMapper, the researcher imports the recordings that make up a session in gazeMapper.
5. *Processing recordings*. Once imported, the researcher launches various tasks in gazeMapper, such as detection of the ArUco markers in the videos and mapping of eye movement data to planes. Most of these processing tasks are performed without a need for manual intervention; there are only two tasks that require manual actions by the researcher. First, depending on the project configuration, the researcher must use a video coding GUI (Fig. 8A) to manually indicate which intervals in each recording contain trials, glassesValidator validations episodes, or episodes used for synchronization of eye tracker data to the scene video camera. If trials or synchronization transients are configured to be coded automatically, the researcher does not have to code these manually but must verify their accuracy and correct them if necessary in the video coding GUI before recording processing can continue. Second, if the project is set up for synchronization of eye movement data to the scene video camera, the researcher must manually check for synchronization using an interface (Fig. 8B) that shows the fixation target and gaze position on the scene camera. If the two signals are found not to be synchronized, the researcher can use this interface to achieve synchronization by dragging the gaze signal to align with the fixation target movement. Processing a recording session with gazeMapper may take between minutes and an hour, depending on the duration of the session.
6. *Result collation*. Once processing of a session has finished, gaze mapped to one or multiple planes can be exported to a single file per participant, along with videos showing gaze from one or multiple participants and a file collecting data quality for all selected recording sessions.

### gazeMapper output and further analysis

gazeMapper provides the following output when a recording session is fully processed:

1. Synchronized gaze data from one or multiple participants mapped to one or multiple planes. Exported gaze data using the results collation functionality of gazeMapper is stored in a single file per participant that contains gaze position in plane coordinates (usually in mm), along with a timestamp (in the time of the reference recording if the session consists of multiple recordings) and a trial number.
2. If included in the study setup, gazeMapper also provides a file with data quality for all the recordings that were selected during export. This file contains, per validation episode, the data quality measures (accuracy and precision) derived using glassesValidator’s (Niehorster et al., 2023) procedure.
3. If selected by the researcher, gazeMapper can produce a gaze overlay video showing the scene video of the participant with their gaze positions overlaid on it.
4. If selected by the researcher, gazeMapper will furthermore produce advanced gaze videos showing gaze mapped to the plane(s), also for the other participants in the same session.

Depending on the specific research question, the researcher would likely want to conduct further analysis with gazeMapper’s output. For instance, one may wish to classify fixations (Hooge et al., 2022b; Andersson et al., 2017; Hein and Zangemeister, 2017) in the gaze data and use these in an Area of Interest (AOI) analysis (Hessels et al., 2016; Holmqvist et al., 2011; Goldberg and Helfman, 2010). Since gazeMapper delivers gaze positions in the world just like many screen-based eye trackers (here the plane represents the computer screen the eye tracker is attached to), these further analysis steps can now be approached using standard analysis methods built for screen-based eye trackers. Such further analyses are not provided by gazeMapper, but many tools addressing these use cases are available (see Niehorster et al. , 2025, for an overview).

## Discussion

We have presented gazeMapper, a tool for the automated mapping of data from one or multiple simultaneously acquired wearable eye tracker recordings to one or multiple planes in the world. We used fiducial markers, specifically ArUco markers, to enable the mapping of head-referenced eye movement data to world-referenced gaze data. Early versions of gazeMapper have already been used for several studies (Hessels et al., 2023, 2024, 2025a) and the same logic for mapping gaze to planes has been used by us for further studies (Niehorster et al., 2020a, 2023; Hessels et al., 2022). While the fiducial approach is commonly used to map gaze to the world (besides the aforementioned studies, see also, e.g., Duchowski et al., 2020; Tabuchi and Hirotomi, 2022; Faraji et al., 2023; Bykowski and Kupiński, 2018, Yang and Chan, 2019; De La Hogue et al., 2024), it should be noted that this approach may not be suitable for all studies. There is evidence that in some settings (e.g., pilots operating an aircraft, Ayala et al., 2024), visible fiducial markers may interfere with the participants’ visual tasks. To mitigate this, markers have been developed that appear black in visible light and are thus not visible to participants, but reveal an ArUco marker under infrared lighting (Ayala et al., 2023; see also, Dogan et al., 2022).

From our experience, what are the pitfalls to avoid when using gazeMapper? There are several reasons why gazeMapper may fail. Many of these are shared by gaze mapping approaches using fiducial markers in general, but not all. Reasons for failure relate to the setup of the study and to participant behavior. Setup-related issues include the following:

1. The array of ArUco markers is attached to a non-flat surface (e.g., a curved monitor). While some tools are designed to handle such situations (e.g., De La Hogue et al., 2024), gazeMapper currently only supports flat surfaces.
2. Fiducial markers are not unique, but the same marker is used in multiple places in the world. This may, for instance, occur if a marker is used in more than one plane, but also if an individual marker used for trial segmentation or recording synchronization is also used for defining a plane. A fiducial marker may furthermore be visible in multiple locations if there are mirrors or other reflective surfaces in the setup or when a live view of the feed of an overview or scene camera is visible.
3. Too few markers are detected for robust mapping of gaze to the plane. Based on experience, for reliable mapping we recommend that at least three markers should be detected for a plane and that these markers should not be very close together. Pose with respect to a single marker is ambiguous and thus cannot be reliably resolved (Elmo Kulanesan et al. , 2024). We cannot give more specific advice on what marker layouts should conform to since this will highly depend on factors such as lighting conditions and camera quality. The researcher is strongly recommended to pilot their setup and task for robust operation.
4. Markers in the camera view are too small or too large for reliable detection (Romero-Ramirez et al., 2019).
5. The size, position or orientation of the markers has not been correctly specified in the plane setup.
6. Lighting conditions prevent marker detection. While in our experience very dark or very light scenes may not be an issue (especially when a large number of markers is visible), non-uniform lighting of a marker may prevent detection. Examples of this are strong shadows, or reflections of overhead lighting on display surfaces such as tablets.

Participant-related issues include the following:

1. Motion blur interfering with marker detection. The scene camera of the mobile eye tracker may undergo fast movement due to excessive participant head movement, which may lead to significant motion blur in the recorded video. Depending on the configurability of the camera used and the flexibility of the setup, it might be possible to mitigate such issues by increasing the illumination of the setup and reducing the exposure time of the camera. Other solutions may consist of improving the robustness of the ArUco detector for motion blur (Romero-Ramirez et al., 2021) or using different fiducial markers that are more robust to motion blur such as RainbowTags (Egri et al., 2022). Neither is currently supported by gazeMapper due to a lack of support in OpenCV, the computer vision library that gazeMapper uses.
2. ArUco markers can only be detected when they are fully in view of the camera and when there is no occlusion. Participant behavior may cause markers to partially fall outside the view of the camera or may cause occlusion (e.g., placing hands or arms over the markers). The impact of such behavior may be mitigated by using many markers to define a plane (see, e.g., Fig. 5B) so that the chance is large that a sufficient number of markers is fully visible most of the time. Another potential solution would be to use markers that are robust to occlusion, such as fractal ArUco markers (Romero-Ramirez et al., 2019) or recursive AprilTags (Krogius et al., 2019). However, neither is currently supported by gazeMapper due to a lack of support in OpenCV, the computer vision library that gazeMapper uses.

The reader may notice that we do not give precise guidelines for mitigating most of these issues. Indeed, whether they crop up in a given recording is highly dependent on the specifics of both the setup and the task that participants perform. We therefore wish to underscore that it is critical for a successful wearable eye-tracking experiment to pilot the setup, the participants’ tasks, and the analysis pipeline. As an example, we briefly highlight the issues that were uncovered and resolved during piloting a recent series of studies using an early version of gazeMapper (Hessels et al. , 2023; Hessels et al. , 2024; Hessels et al. , 2025a; see Hessels et al. , 2025b, for further discussion). Note that these relate closely to the setup-related and participant-related issues listed above. First, we had to ensure that enough markers on the table were visible, even after occlusion of some of the markers by the hands, heads, and/or upper bodies in the eye tracker scene cameras or overview camera (see Fig. 5A for the resulting set of markers). Second, we found out during piloting that there was overlap between the ArUco markers used on the table and on the glassesValidator poster used during eye tracker validation. This was solved by ensuring the glassesValidator poster was never in view of the cameras during the experiment. Third, there were substantial reflections from the overhead lighting in the overview camera for early versions of the setup, making, among other issues, ArUco markers difficult to detect. The setup was modified to minimize such overhead reflections. Fourth, we piloted the exposure setting of the Pupil Invisible scene camera to optimize both ArUco marker detection and face detection. This meant striking a balance between short exposure durations with little motion blur for ArUco marker detection, and longer exposure durations with more motion blur for face detection. Finally, we piloted many settings for the overview camera (white balance, exposure duration, recording frequency, etc.) as well as the temporal accuracy of the synchronization between the overview camera and eye tracker scene cameras. We expect that many of the issues we encountered would apply to other studies as well.

### Future directions

The overall aim of developing tools such as gazeMapper, glassesValidator (Niehorster et al., 2023, for determining data quality of wearable eye tracker recordings), glassesViewer (Niehorster et al., 2020, for accessing and event coding of data from Tobii Glasses 2 and 3 wearable eye trackers) and GazeCode (Benjamins et al., 2018, for per-fixation manual mapping of wearable eye tracker data) is to make robust methods for the analysis of wearable eye tracker data available to all researchers, also those who do not have the skills or interest to program an analysis pipeline themselves. While many different analysis methods for wearable eye-tracking data have been published over the last decades (c.f., Niehorster et al., 2025; and also Fu et al., 2024), very few of these tools are easily usable because most either require significant computer and programming skills to get provided code to run, or because the publication only described the method but no implementation is provided at all. gazeMapper currently only covers a small section of the wealth of available methods, and there are several interesting opportunities for extension that may be explored in the future:

1. An extension to support non-flat objects defined by fiducial markers, such as would be used for cockpits and nuclear power plant control panels (Langstrand et al., 2018; Duchowski et al., 2020; De La Hogue et al., 2024), and curved computer screens.
2. Head-tracking using an external camera. As identified above, participant movement may induce motion blur in the video captured with the head-attached scene camera of a mobile eye tracker. It is therefore worthwhile to explore using an external camera for determining head pose in the world, for the purpose of mapping eye movements to the world. Such an external camera, because it is not constrained by the size and power consumption limits that a wearable camera must adhere to, can provide vastly superior images of the moving head (see, e.g., Hooge et al. , 2024), potentially much reducing the motion blur issue. It, however, also introduces the new problem of transforming gaze data to the reference frame in which head pose is acquired, taking into account the different rotation axes of the eye and the head (Allison et al., 1996; Johnson et al., 2007; Ronsse et al., 2007; Cesqui et al., 2013). An alternative solution might be mounting an additional higher-quality front-facing camera on the head (e.g., Kothari et al. , 2020; Shankar et al. , 2021; DuTell et al. , 2024). This would, however, also require an additional transformation between the reference frames of the eye tracker and the additional camera.
3. An extension to perform area of interest (AOI) analysis on the world-referenced gaze data (e.g., De La Hogue et al. , 2024; Duchowski et al. , 2020). While gazeMapper currently already outputs world-referenced gaze data, which greatly simplifies AOI analysis, some basic programming skills (or extreme perseverance) are still required to perform such analyses. Built-in support for defining AOIs on the planes in the world and using these to perform AOI analyses (Holmqvist et al., 2011; Hessels et al., 2016) would further improve the usefulness of the tool.

Since gazeMapper is planned to undergo further development, potentially including in the areas identified above, we recommend that the reader monitor gazeMapper’s repository on GitHub (https://github.com/dcnieho/gazeMapper) to keep abreast of future developments. Contributions by the reader, such as support for further eye trackers, are also warmly welcomed.

## Open practices statement

The gazeMapper tool is available at https://github.com/dcnieho/gazeMapper.

## Data Availability

The gazeMapper tool is available at https://github.com/dcnieho/gazeMapper.

## Footnotes

1 It should be noted that gazeMapper uses the cv2.aruco library from OpenCV for ArUco marker detection. This library also supports April tags (Wang and Olson, 2016; Krogius et al., 2019), and as such April tags can be used with gazeMapper without needing to modify its code.

## Declarations

### Code availability

The gazeMapper tool is available at https://github.com/dcnieho/gazeMapper.
